# Shear wave cardiovascular MR elastography using intrinsic cardiac motion for transducer-free non-invasive evaluation of myocardial shear wave velocity

**DOI:** 10.1038/s41598-020-79231-z

**Published:** 2021-01-14

**Authors:** Marian Amber Troelstra, Jurgen Henk Runge, Emma Burnhope, Alessandro Polcaro, Christian Guenthner, Torben Schneider, Reza Razavi, Tevfik F. Ismail, Jordi Martorell, Ralph Sinkus

**Affiliations:** 1 School of Biomedical Engineering and Imaging Sciences, King’s College London, London, UK; 2 Department of Radiology and Nuclear Medicine, Amsterdam UMC, University of Amsterdam, Amsterdam, The Netherlands; 3 Department of Cardiology, Guy’s and St Thomas’ NHS Foundation Trust, London, UK; 4 Department of Chemical Engineering and Material Sciences, IQS School of Engineering, Universitat Ramon Llull, Via Augusta 390, 08017 Barcelona, Spain; 5 Institute for Biomedical Engineering, University and ETH Zurich, Zurich, Switzerland; 6 Philips Research, Hamburg, Germany; 7 Philips, Guildford, UK; 8 Inserm U1148, LVTS, University Paris Diderot, University Paris 13, Paris, France

**Keywords:** Cardiology, Translational research, Cardiovascular diseases, Magnetic resonance imaging

## Abstract

Changes in myocardial stiffness may represent a valuable biomarker for early tissue injury or adverse remodeling. In this study, we developed and validated a novel transducer-free magnetic resonance elastography (MRE) approach for quantifying myocardial biomechanics using aortic valve closure-induced shear waves. Using motion-sensitized two-dimensional pencil beams, septal shear waves were imaged at high temporal resolution. Shear wave speed was measured using time-of-flight of waves travelling between two pencil beams and corrected for geometrical biases. After validation in phantoms, results from twelve healthy volunteers and five cardiac patients (two left ventricular hypertrophy, two myocardial infarcts, and one without confirmed pathology) were obtained. Torsional shear wave speed in the phantom was 3.0 ± 0.1 m/s, corresponding with reference speeds of 2.8 ± 0.1 m/s. Geometrically-biased flexural shear wave speed was 1.9 ± 0.1 m/s, corresponding with simulation values of 2.0 m/s. Corrected septal shear wave speeds were significantly higher in patients than healthy volunteers [14.1 (11.0–15.8) m/s versus 3.6 (2.7–4.3) m/s, p = 0.001]. The interobserver 95%-limits-of-agreement in healthy volunteers were ± 1.3 m/s and interstudy 95%-limits-of-agreement − 0.7 to 1.2 m/s. In conclusion, myocardial shear wave speed can be measured using aortic valve closure-induced shear waves, with cardiac patients showing significantly higher shear wave speeds than healthy volunteers. This non-invasive measure may provide valuable insights into the pathophysiology of heart failure.

## Introduction

Cardiovascular magnetic resonance (CMR) imaging has repeatedly demonstrated clinical utility for the diagnostic and prognostic evaluation of heart failure patients with both ischemic^1^ and non-ischemic etiologies^2,3^. It is widely regarded as the gold-standard for the assessment of chamber volumes and systolic function and readily allows non-invasive tissue characterization without the need for ionizing radiation^4,5^. Changes in tissue composition alter the biomechanical properties of myocardium and thereby engender lusitropic and contractile abnormalities which serve as a potent substrate for heart failure^6,7^. Myocardial stiffness is increased in patients with heart failure with preserved ejection fraction^8–10^, hypertrophic cardiomyopathy^11^, and myocardial ischemia^12^. It may therefore serve as a valuable biomarker for early tissue injury, as well as for conditions where abnormalities in diastole predominate, however, myocardial stiffness is not readily measured non-invasively.

Magnetic resonance elastography (MRE) may constitute a means of addressing this unmet need and currently is in clinical use for staging liver fibrosis and inferring tumor characteristics in the breast amongst other applications^13,14^. Elastography techniques aim to determine intrinsic biomechanical properties of the tissue using the propagation of shear waves, induced by either intrinsic or external mechanical wave generation. As tissue is quasi-incompressible, most elastography methods focus on the quantification of the shear modulus, which characterizes the mechanical integrity of the solid tissue component^13^. Stiffness values are typically reconstructed using shear deformations caused by the waves, solving specific equations based on the chosen tissue model^15^.

Cardiovascular elastography has proven to be challenging due to several factors. Firstly, the combination of a relatively high stiffness, comparable to large muscles such as the biceps^16,17^ and reduced thickness of the ventricle, leads to complex wave patterns. Secondly, the tissue exhibits anisotropic mechanical properties, and requires compensation for substantial cardiac and respiratory physiological motion during the cardiac cycle. Several in vivo ultrasound elastography studies describe shear wave propagation of naturally generated shear waves in the interventricular septum^17,18^. While myocardial stiffness can be estimated, these studies are strongly dependent on favorable intercostal windows, and depth measurement is constrained due to the limited penetration of ultrasound in tissue^19^. Cardiovascular MRE using an external mechanical wave source is complicated due to the high stiffness of myocardial tissue, requiring high vibrational frequencies to produce waves with a sufficiently short wavelength for reliable quantification. In turn, high vibrational frequencies lead to increased attenuation of the waves due to the underlying frequency power-law^20^, rendering full cardiac insonation with sufficient wave amplitude difficult^21^. Additionally, the need for extra hardware for mechanical wave generation complicates clinical implementation. Despite these obstacles, some interesting in vivo cardiac MRE studies in animals^22–27^ and in human subjects^28–32^ have been produced.

To overcome the challenges presented by cardiovascular MRE using externally generated waves, we have developed a novel cardiac pencil beam sequence to determine myocardial shear wave velocity using intrinsic shear wave propagation induced by aortic valve closure. A 2D pencil beam sensitized to motion via a bipolar encoding gradient permits imaging of displacements in any spatial direction along the symmetry axis of the pencil beam at time-lapses as short as ~ 20 ms. The aim of this study is to provide proof-of-concept for using this novel MRE method, whereby pencil beam shear wave elastography is implemented to determine the shear wave velocity of the myocardium using aortic valve closure as an intrinsic wave source. After discussing the theory of wave propagation in a hollow cylinder, we show in corresponding phantom experiments that the proposed pencil beam method yields propagation speeds for the torsional and flexural wave modes that match theoretical expectations. Next, we show its application in healthy volunteers and provide initial results for patients with cardiovascular pathology.

## Methods

### Theory & simulations

#### Geometry

**Figure 1:**
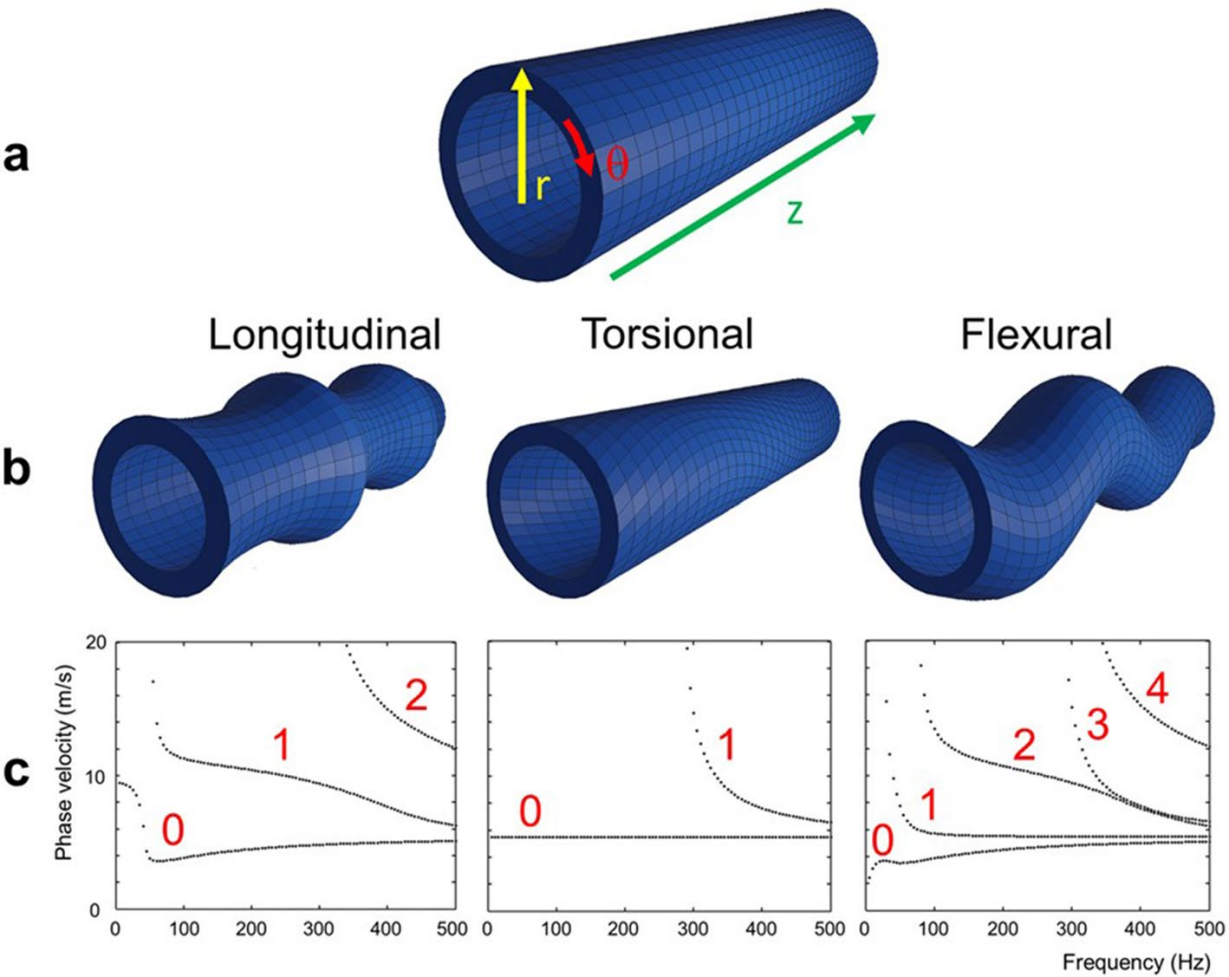
Different wave modes in a hollow cylindrical structure. **(a)** Geometrical displacement directions of a wave in a cylinder. The green arrow describes the axial displacement in the *z*-direction; the yellow arrow radial displacement; and the red arrow circumferential displacement. (**b)** Visualization of displacement behavior for longitudinal, torsional, and flexural modes in a hollow cylinder, respectively. (**c)** The phase velocities of the 3 fundamental modes (left to right: longitudinal, torsional, flexural) and their higher order modes in the bandwidth from 0 to 500 Hz for a cylinder of 30 mm inner radius, 10 mm wall thickness, shear velocity √30 m/s, longitudinal velocity 1550 m/s, density 1000 kg/m^3^. The lowest order torsional mode is non-dispersive and hence propagates at a speed reflecting the true material properties independent of the frequency and the geometry.

The geometry of the heart is complex but, for simplicity, the left ventricle can be approximated by a hollow cylinder. A mechanical wave that propagates within the cylinder wall in the axial direction (z-axis) can be described by three generic directions of motion: longitudinal, torsional and flexural (Fig. 1a,b)^33^. The finite geometry of the cylinder and the small thickness of the wall compared to the wavelength leads to wave guidance effects, *i.e.*, the waves are geometrically dispersive^33^. This means their speed is frequency-dependent as the wave is confined within a structure much smaller than the wavelength. The higher the frequency, the less the apparent shear wave speed is biased, since the wavelength becomes shorter and the geometrical constraints less important.

#### Displacement behavior of different wave modes

Longitudinal modes describe axially symmetrical motion, with displacement components both in the axial and radial direction. Torsional modes describe motion that is entirely polarized in the circumferential direction and is observable as a uniform clockwise or counterclockwise displacement throughout the whole cross-section of a cylinder. Flexural modes consist of a combination of motion in all three directions (radial, axial and circumferential), resembling the movement of a snake and observable as a uniform in-plane displacement of the whole cross-section^33^.

Theory states that the velocity of shear wave propagation depends on the material’s properties, wall thickness, and the radius of the structure for dispersive modes within a hollow geometry such as the ventricle^34^. In general, a mode is evanescent and hence non-propagative if the wave frequency is below the so-called cut-on frequency of that mode. The aortic valve closes within a time frame or period of ~ 5 ms or less^35^. This can be seen as a sinusoidal oscillation occurring within this timeframe, leading to a wave with an expected central frequency around 200 Hz (1/0.005 s), zero energy at 0 Hz, and contributions at higher frequencies. This is supported by in vivo acoustic measurements showing peak frequencies between 200 and 300 Hz for aortic valve closure^36–38^. For the bias calculus we therefore selected the frequency range from 200 to 300 Hz as the representative bandwidth. The expected dispersion curves for the three wave modes were obtained using the analytic software package GUIGUW^34^ (Fig. 1c). Simulations were performed in the bandwidth from 0-500 Hz for a cylinder of 30 mm inner radius, 10 mm wall thickness, shear velocity √30 m/s, longitudinal velocity 1550 m/s and density 1000 kg/m^3^.

#### Torsional mode

Within the torsional modes, the fundamental mode propagates at a shear wave velocity that is independent of the wave frequency and is thus non-dispersive (Fig. 1c). Hence, its value is truly representative for the shear properties of the material. The superior modes are strongly dispersive, but only start propagating after a cut-on frequency that can be calculated analytically based on the stiffness, the radius, and wall thickness of the structure^33^. Assuming a normal LV geometry (inner radius 30 mm, septal thickness 10 mm, shear wave speed 5.5 m/s), the cut-on frequency for the first higher torsional mode is ~ 300 Hz, with the cut-on frequency rising when the wall thickness is reduced. Hence, when imaging the torsional mode, the obtained results would mainly originate from the non-dispersive fundamental mode that can be considered pure and thus unbiased.

#### Flexural and longitudinal modes

Conversely, the fundamental modes of both the flexural and longitudinal modes are dispersive and additional modes besides the fundamental mode exist within the expected 200 Hz frequency range. Assuming the same geometry and material shear speed as mentioned above, the fundamental longitudinal and flexural modes travel at very similar speeds of ~ 4.5 m/s in the expected 200 Hz frequency range, while the additional modes can propagate at higher speeds.

For the flexural modes, our selected frequency bandwidth sums three components; therefore, we expect to see two slow modes with very similar speeds separated from a much faster third mode (Fig. 1c). The fast component easily separates from the slow components in time. Ultimately, the two slowest flexural modes converge towards the intrinsic shear speed at higher frequencies and hence start to become unbiased. Therefore, when analyzing the space–time diagrams of travelling waves, one should focus on the trailing edge to select the slowest wave component and correct its bias if the geometry is known.

For the longitudinal motion, two modes co-exist (the fundamental and the first mode) in the frequency range that we are interested in, but their speeds are considerably different (Fig. 1c). The first mode travels at a speed more than twice the speed of the fundamental and thus we would expect them to separate in time. This can be further mitigated by targeting the trailing edge in the space–time diagram analysis as explained above.

### Phase and group velocity

Measuring a unique shear wave velocity using a wave comprised of a range of frequencies in a dispersive system is challenging. Previous studies have shown that group velocity provides different results from phase velocity^16^. By choosing the trailing edge to determine shear wave speed as explained above, we ensure we select the lowest and hence slowest mode in our range of frequencies. For the flexural wave (Fig. 1c), this consists of two modes that travel at similar velocities and show limited dispersive behavior in the frequency range from about 200–300 Hz. Therefore, by choosing the trailing edge, we try to minimize the effect of group versus phase velocity in our analysis.

#### Wave mode of aortic valve closure

We assumed that mechanical excitation originating from aortic valve closure is predominantly in the z-direction. The fundamental mode for a longitudinal excitation (Fig. 1c, left, “0”) is flexural in direction and can therefore be selected by properly orienting the motion encoding gradient within the sequence.

### Concept of cNAV-tMRE

#### Data acquisition

To quantify the shear wave propagation speed, we modified a two-dimensional pencil beam sequence commonly used as a respiratory navigator. The method is called “cardiac NAV transient MRE” (cNAV-tMRE), to distinguish it from previous transient MRE approaches that relied on the exact timing of aortic valve closure^39^. This previous approach provided only a snapshot of the propagating wave, from which solely wavelength could be extracted and not shear wave speed. The newly proposed cNAV-tMRE method overcomes these limitations, providing a highly resolved time series (0.3 ms) of one-dimensional images over the entire cardiac cycle from which shear wave speed can be determined.

**Figure 2:**
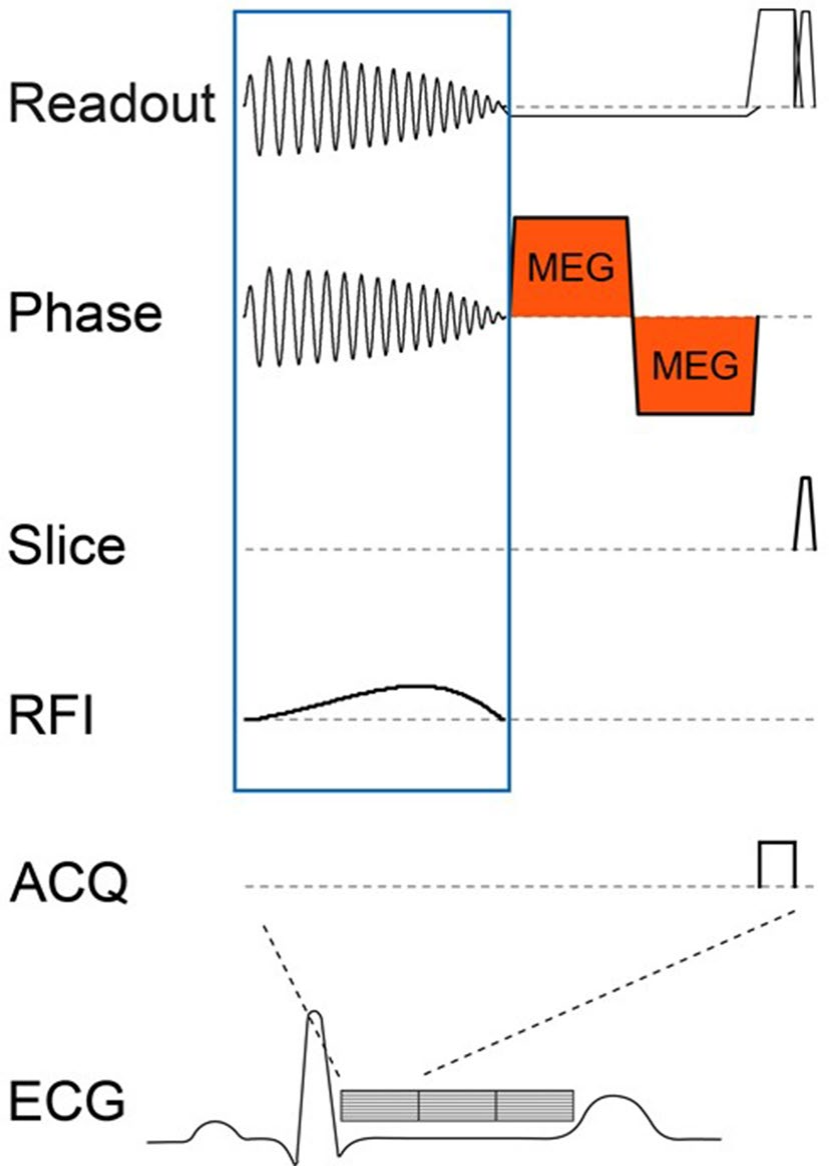
Pulse sequence diagram of 2D pencil beam navigator. The motion sensitized 2D pencil beam navigator (NAV) consists of the 2D excitation block (readout, phase and radiofrequency impulse = RFI) spatially selecting a cylindrical volume (blue box), a bipolar motion-encoding gradient (MEG) attached to one of the 3 spatial directions (orange, shown here for the phase direction), and a short readout during which the signal is collected (ACQ). The entire duration for one NAV is about 20 ms. Therefore, throughout one cardiac cycle of approximately 1000 ms duration, roughly 40 NAV’s can be shot.

A cylindrical motion-sensitized pencil beam volume of a user-determined diameter (default: 30 mm) is generated by a two-dimensional pencil beam excitation pulse, followed by a motion-encoding gradient, and a read-out gradient (Fig. 2). After Fourier transformation, the phase of the pencil beam signal yields a one-dimensional line-image that depicts motion in the direction of the motion-encoding gradient, similar to classical MRE. Importantly, motion sensitization can be achieved in any direction, which is crucial as it allows the assessment of individual normal modes.

**Figure 3:**
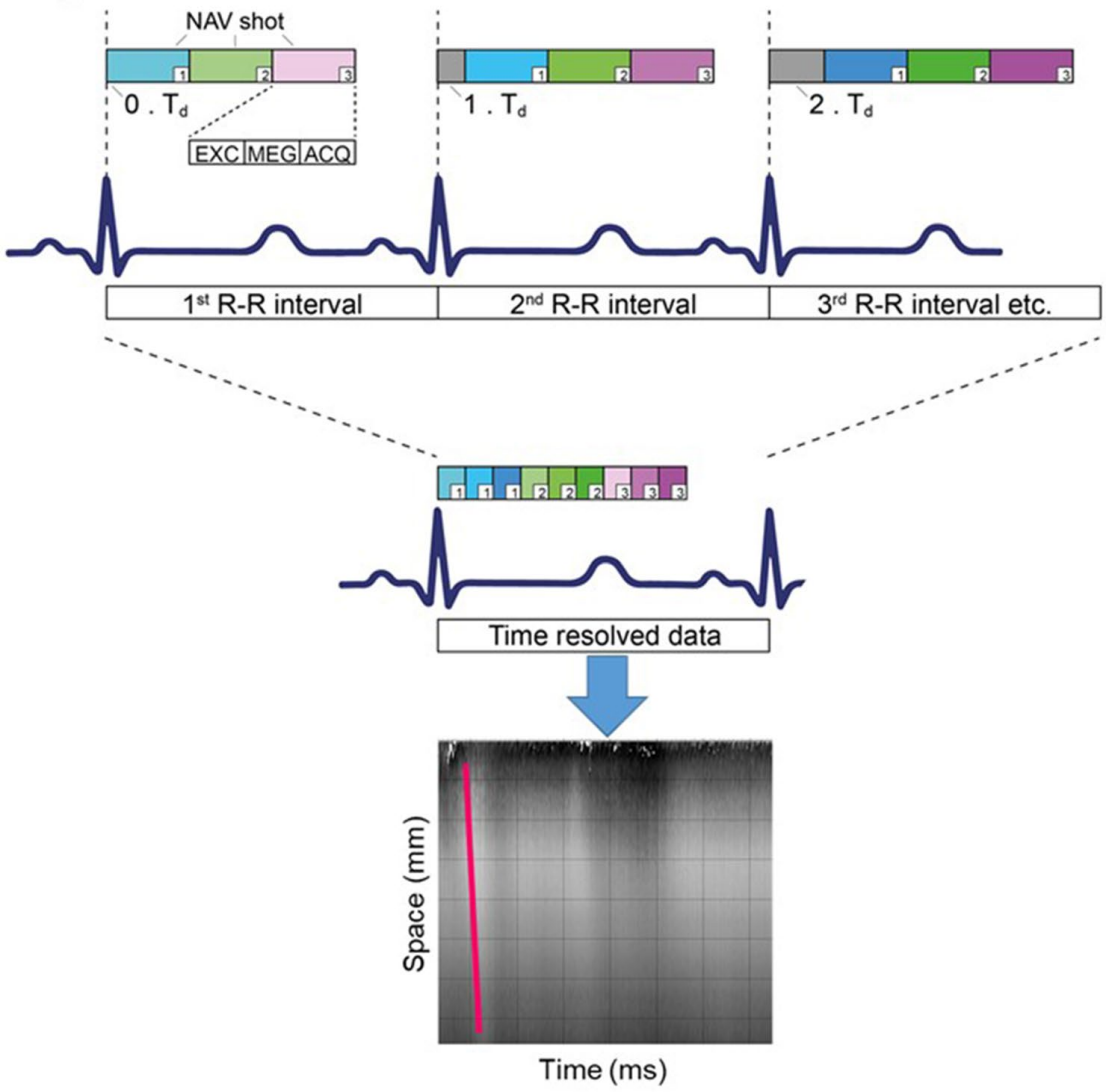
Image acquisition and data re-sorting. Multiple cNAV shots (~ 40) are acquired during each heartbeat. For each subsequent heartbeat, an additional temporal delay T_d_ is introduced (grey area) to shift the NAV acquisition relative to the closure of the aortic value. Data are re-sorted afterwards according to their temporal position relative to the R-wave. This allows to obtain temporal resolutions up to 0.3 ms, two orders of magnitude below the duration of each NAV (20 ms). *EXC* excitation pulse, *MEG* motion-encoding gradients, *ACQ* readout acquisition.

The same volume is repeatedly excited throughout multiple cardiac cycles with a total duration for a single shot of approximately 20 ms. High temporal resolution is achieved by time-shifting the data acquisition in subsequent cardiac cycles with respect to the R-wave of the vectorcardiogram (VCG)^40^. After data acquisition, the magnitude and phase image lines are re-sorted according to their temporal position in relation to the R-wave, resulting in a time-resolved space–time waterfall plot (Fig. 3). Depending on the total number of measured cardiac cycles, temporal resolutions of ~ 0.3 ms can be achieved within an acceptable breath hold length (15 cardiac cycles within a single breath hold) for a total of four breath holds (20 ms/(4 breath holds × 15 cardiac cycles) = 0.333 ms). This paper bases the wave speed on the flexural mode, as initial in vivo imaging showed that motion encoding for the flexural mode provided the highest signal amplitude with the clearest phase perturbation and therefore the most reproducible results.

#### MRI parameters

All data was acquired on a Philips 3T Achieva MRI scanner (Philips, Best, the Netherlands). Phantom data was acquired using a surface coil and in vivo data using a 16-channel phased-array anterior coil and a 10-channel phase-array posterior coil. The cNAV-tMRE sequence was acquired using a pencil beam volume with a standard diameter of 30 mm and length 80 mm. The repetition time was 20.2 ms, echo time 9.21 ms and flip angle 15 degrees.

#### Positioning of the pencil beam

**Figure 4:**
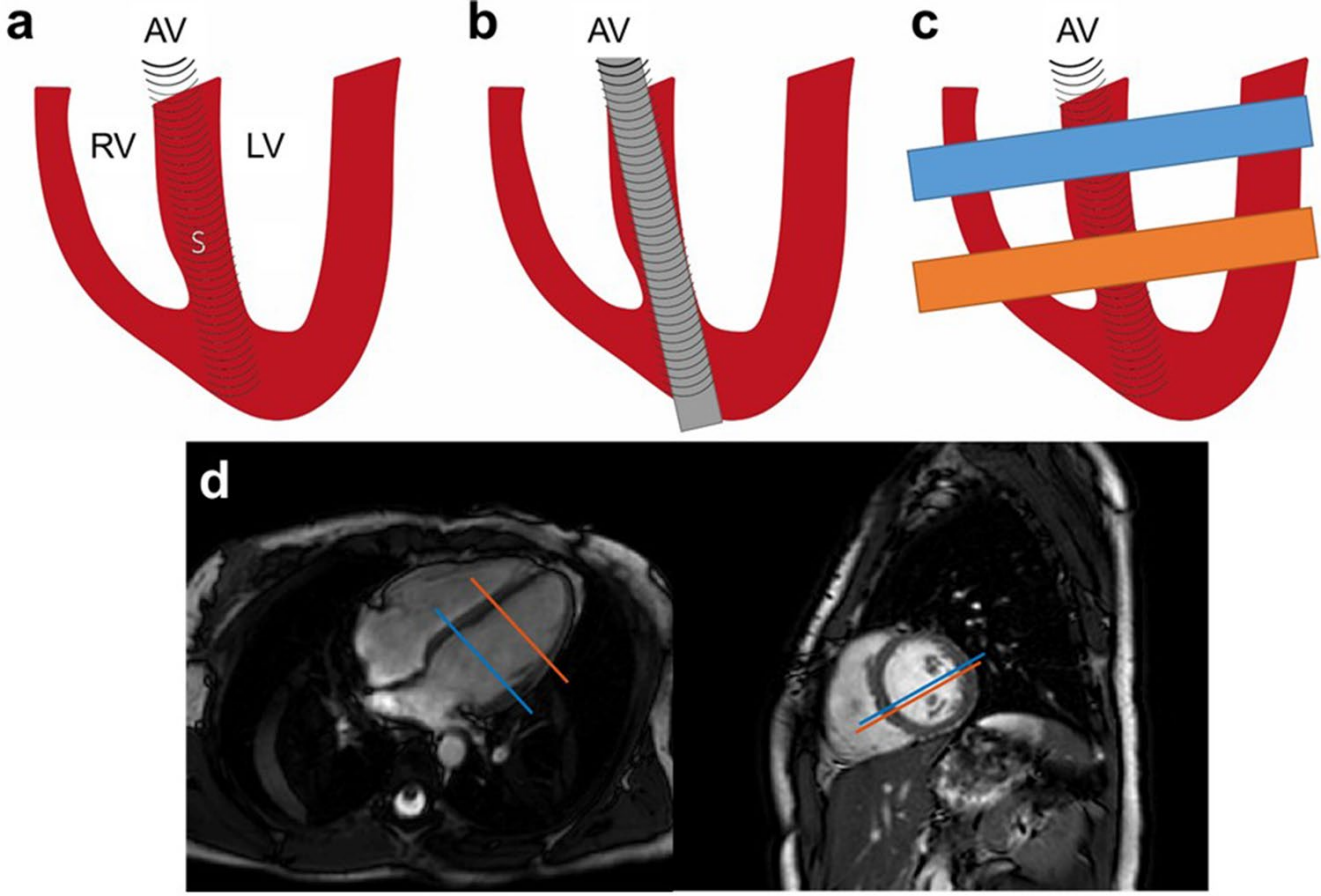
cNAV positioning in vivo. **(a)** Conceptually, the closure of the aortic valve will lead to mechanical shear waves that propagate from apex to base (top down). *AV* aortic valve, *RV* right ventricle, *LV* left ventricle, *S* septum. (**b)** The 2D volume of the pencil beam was therefore initially positioned within the interventricular septum in apex-base direction. (**c)** Positioning with two pencil beam volumes perpendicular to the interventricular septum, one at the basal and one at the apical septum, solved issues with image analysis caused by partial volume effects and motion. (**d)** Example of in vivo positioning in a healthy volunteer.

Initially, our idea was to position the pencil beam’s z-axis parallel to the direction of propagation of the shear wave, which in the phantoms results in positioning it in line with the wave source and in vivo within and parallel to the interventricular septum (Fig. 4a,b). This method uses a single parallel pencil beam. Similar to the ultrasound-based approach from Echosense for quantifying liver stiffness^41^, the slope of the propagating wavefront yields the measured apparent speed of the selected shear wave mode. While this approach is robust when little to no coarse motion occurs (*i.e.*, in a phantom), in vivo applications are liable to patient motion and its associated partial volume effects. Bulk motion between initial planning and scanning causes the interventricular septum to (partially) move out of the pencil beam volume during the cardiac cycle. Under these circumstances, the spatial averaging effect originating from the 1D read-out, whereby the signals are radially collapsed onto a single point as a function of z, diminishes the signal truly originating from the myocardium. In some cases, this made it impossible to accurately identify a propagating wave in the phase waterfall plot due to noise from the blood pool.

To overcome this effect in vivo, we implemented a time-of-flight approach with two pencil beams positioned perpendicular to the interventricular septum (Fig. 4c,d), ensuring the full width of the septum is always in the field-of-view. By measuring at two different locations with a known distance between each, shear wave speed can be calculated using a time-of-flight method, where the distance between pencil beams is divided by the difference between the arrival time of the shear wave at the first pencil beam volume (proximal to the wave source) and the second pencil beam volume (distal). The first pencil beam is positioned at the basal septum, while the second is placed at the midventricular septum, approximately 25–35 mm towards the apex. This planning ensured the pencil beam volumes were located within the myocardial septum, while allowing enough distance for measuring shear wave velocity. With a temporal resolution of 0.33 ms, the minimal distance between the two volumes for being able to determine shear wave velocity would be 4.5 mm at a shear wave velocity of 15 m/s. This approach demonstrated extreme robustness against patient motion and facilitated reproducible scan planning.

#### Shear speed reconstruction

All images and data were processed using in-house built Matlab software (Matlab R2015b, Mathworks, Natick, MA, USA). Consecutive 1D phase images of the pencil beam navigator are locally unwrapped in the space–time domain before determining shear wave speed^42^. Temporal artefacts in the phase images, originating from an imperfect reproduction of the initial breath hold position, are filtered out using a 2D Fourier transform-based filtering algorithm. Propagating shear waves are then seen as phase perturbations within the waterfall diagram. In the case of a single navigator, the inclination of the perturbation in the waterfall diagram directly yields the apparent wave speed. With the double navigator time-of-flight approach, their separation in space divided by the measured time delay between the time points when the wave passes through each navigator, provides an estimate of the shear wave speed. In both cases, it is necessary to determine a phase perturbation as a function of time. We use the maximum temporal phase derivative as the assumed position of the wave within a temporal profile.

#### Phantom measurements

A homogeneous rectangular (30 × 60 × 80 mm^3^ [H  ×  D  ×  W]) block of plastisol (Lure Flex, Lure Factors, Doncaster, UK) with non-dispersive material properties was used for a classical MRE experiment performed at a continuous mechanical excitation of 140Hz^43,44^. This served as a reference for the phantom’s true material properties, as wave guidance effects can be ignored as the shear wavelength at this frequency (~ 1 cm) is significantly smaller than the dimensions of the object. Furthermore, as the material is non-dispersive, it is not important that the frequency of 140 Hz does not match the bandwidth established for in vivo experiments, i.e., 200 Hz-300 Hz. This was confirmed by comparing classical MRE to the cNAV-tMRE sequence in a gel exhibiting a shear wave velocity of approximately 1 m/s, which showed identical propagation speeds for varying excitation frequencies between 100 and 400 Hz (Supplementary Fig. 1).

The plastisol material block was then used for a transient elastography experiment where the mechanical push had a central frequency of 140 Hz using the new cNAV-tMRE sequence. Ideally, both experiments should yield the same speeds. Subsequently, the rectangular block was exchanged by a hollow cylindrical phantom made from the same material mimicking left ventricular dimensions. Shear wave speeds for the torsional and flexural modes were measured and compared to predictions based on simulations. Mechanical waves were generated using a custom, in-house built electromagnetically driven push–pull rod transducer.

### In vivo cardiac experiments

Twelve healthy volunteers (ages: 26 to 44 years, male:female 4:8) and five patients (ages: 36 to 71 years, male:female 4:1) with suspected cardiac pathology were included. The first patient had a family history of arrhythmogenic cardiomyopathy and underwent a cardiovascular MRI study for diagnostic screening. This routine clinical MRI scan revealed normal anatomy without any diagnostic criteria for the condition or any other cardiovascular disease. The other patients had confirmed cardiac pathology: one patient with left ventricular hypertrophy secondary to hypertensive heart disease, one patient with left ventricular hypertrophy due to hypertrophic cardiomyopathy, and two patients with previous myocardial infarction. Healthy volunteers were recruited via King’s College advertising, while cardiac patients were recruited via treating cardiologists. All experimental work was carried out in accordance with the principles of the Declaration of Helsinki and approved by the National Research Ethics Service (reference number 1/11/12 for healthy volunteers and 15/NS/0030 for cardiac patients) as well as by the institutional review boards (Guy’s and St Thomas’ NHS Foundation Trust/King’s College London). Written informed consent was obtained from all participants. After obtaining standard cardiac anatomical and cine views, shear wave speed results were obtained with the time-of-flight method using the cNAV-tMRE images acquired at two locations, perpendicular to the ventricular septum (Fig. 4d).

### Reproducibility

Analysis of the cNAV-MRE images requires specific software, and the wave trajectories must be determined from complex datasets, potentially leading to inter-observer variance. To confirm that the results are not biased, two different experienced observers (EB and AP) independently performed the analysis of shear wave propagation for the healthy volunteers to determine the intra-observer variability.

Reproducibility of the cNAV-MRE method within individual subjects was determined to ensure that any differences found between healthy volunteers and patients would not be explained by normal variances of the method. Paired data was collected from a cohort of twelve healthy volunteers. Each volunteer received a total of two scans, both on separate days and using the same MRI scanner, after which interstudy variability was determined.

### Flexural wave bias correction

**Figure 5:**
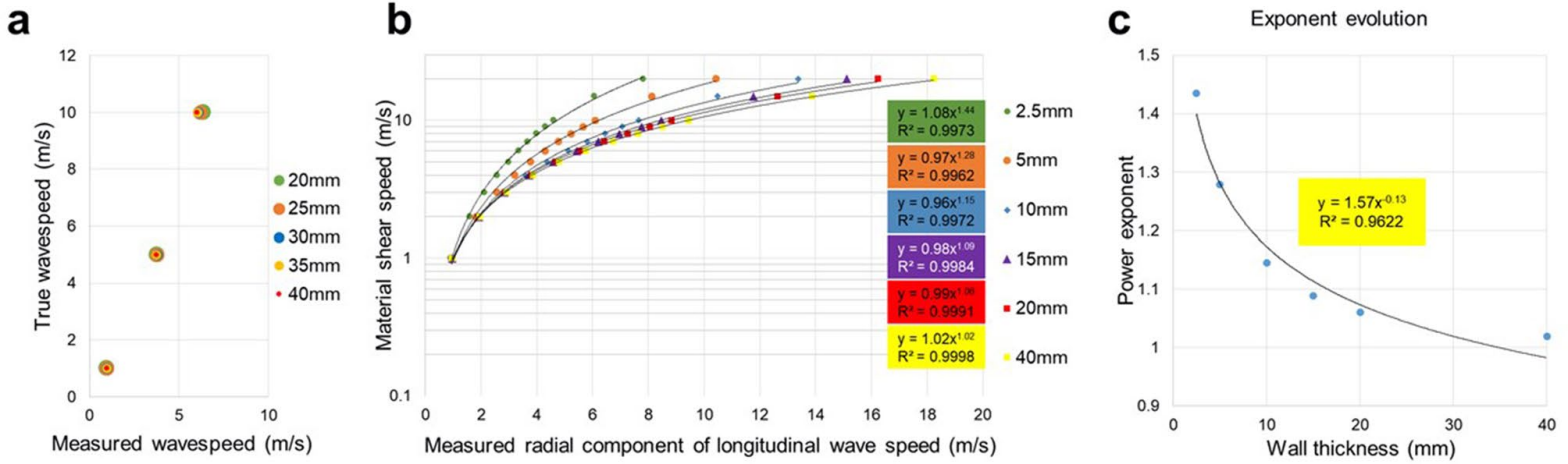
Flexural wave bias correction. **(a)** Analytic results of flexural wave speeds in a hollow cylinder for different material stiffnesses and varying inner cylinder radius given a fixed wall thickness of 5 mm. Clearly, the inner cylinder radius does not significantly impact measured wave speeds. Consequently, to simplify the approach, the inner radius is not included as a factor for the geometrical bias correction. (**b)** Log-linear plot of measured flexural component of the wave speed (*x-axis)* versus the true material shear speed (*y-axis)* for varying wall thicknesses assuming a fixed radius of 30 mm. Dispersion curves can be well approximated by power-law functions where only the exponent varies as a function of wall thickness with a pre-factor very close to one. (**c)** Power-law exponents as obtained in (**b)** as a function of wall thickness follow equally an empirical power-law. Overall, for a given wall thickness and a measured flexural shear wave speed, we can estimate the true shear speed via: α = 1.57*WT^−0.13^, C_true_ = C_flex_^α^, *WT* wall thickness.

As explained in the Theory & Simulations section above, the measured wave speed does not reflect the true underlying material properties but is biased due to geometrical constraints. Since geometrical information such as wall thickness and chamber radius can be extracted from anatomical MRI images, it is therefore possible to correct for any bias. To establish a correction look-up table, we investigated the dependence of apparent shear wave speed for the slowest flexural wave mode originating from a longitudinal excitation with respect to geometrical factors in the frequency range of 200 Hz-300 Hz. Figure 5a shows the dependence of measured and true wave speed for different inner cylinder radii for a fixed wall thickness of 5 mm. It is evident that the inner radius does not significantly impact the measured speed and is consequently ignored for the subsequent analysis. Figure 5b shows the relationship between measured and true wave speed for different wall thicknesses. Here, the inner radius is fixed to 30 mm and the wall thickness modified from 2.5 mm to 40 mm, for varying intrinsic speeds between 1 and 20 m/s. As expected, wall thickness significantly impacts measured shear wave speed. Empirically, we found that the functional relationship between measured ($${c}_{measured})$$ and true shear wave speed ($${c}_{true})$$ is well approximated by a power-law defined in Eq. (1):

1 $${c}_{true}=1.0*{c}_{measured}^{\alpha }$$

The exponent $\alpha$ changes with wall thickness (WT) and in the measured speed range that is reasonable for our study population, we find the heuristic dependency depicted in Fig. 5c and as defined in Eq. (2):

2 $$\alpha =1.57*{WT}^{-0.13}$$

Thus, the true speed can be directly estimated using the wall thickness measured from anatomical cine images around aortic valve closure and shear wave speed measured via our cNAV-tMRE method.

### Statistics

Data are presented as mean ± standard deviation or as median (first quartile-third quartile) for boxplots. Interobserver and interstudy variability were evaluated using the methods of Bland and Altman which were used to derive an estimate of bias and coefficients of repeatability expressed as the 95% limits of agreement^45^. Patient versus volunteer datasets were compared using the Mann–Whitney U test. Two-tailed values of p < 0.05 were considered statistically significant. All data were processed using Matlab R2015b (Mathworks, Natick, MA, USA).

## Results

### Phantom measurements

**Figure 6:**
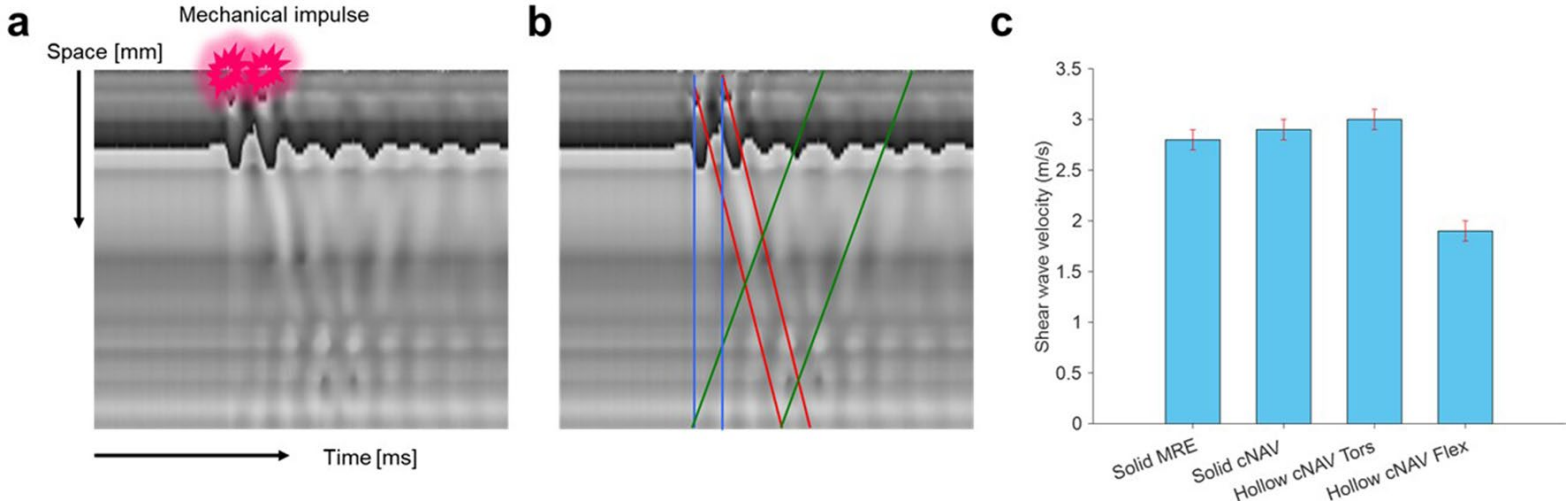
Validation of cNAV method in a phantom material. **(a)** Example of a time-distance waterfall plot obtained in a large solid cylinder showing the phase of the pencil beam acquisition. Two mechanical impulses (red stars) provoke the propagation of different wave modes travelling through the sampled volume over time. (**b)** In blue, propagation of the compressional (longitudinal) wave. Due to its high speed (1550 m/s) this is practically a vertical line. In red, propagation of the shear wave. In green, reflected shear waves and shear waves originating from mode conversion. (**c)** Shear wave velocity values obtained with classical MRE in a large block of plastisol (left) serving as the reference standard, and results obtained with the pencil beam method for different phantom geometries (solid, hollow) and different wave modes (torsional, flexural).

The waterfall diagrams obtained within the large block of plastisol are shown in Fig. 6a,b. Two cycles of mechanical excitation at 140 Hz were generated at the surface of the phantom (red stars in Fig. 6a). This led to the generation of longitudinal (blue) and shear waves (red) propagating in space (vertical) and time (horizontal). Mode conversion of longitudinal to shear and simple wave reflections at the opposite side of the phantom lead to reflected shear waves (green). Due to the high speed of the longitudinal wave (1550 m/s), its propagation appears as an almost vertical line in the waterfall diagram. Clearly, our current temporal resolution is not sufficient to extract any reliable material quantities from that wave. Shear waves, however, demonstrated a prominent and measurable inclination due to their low speed which was of the order of 1–10 m/s.

As seen in Fig. 6c, the reference MRE acquisition yielded a shear wave speed of 2.8 ± 0.1 m/s for the plastisol. The corresponding experiment obtained with the cNAV-tMRE sequence yielded 2.9 ± 0.1 m/s, which agrees well within one standard deviation of the reference value. Transient cNAV-tMRE experiments in the hollow cylinder provided shear speeds of 3.0 ± 0.1 m/s for the torsional mode and 1.9 ± 0.1 m/s for the flexural mode. As predicted based on simulations, the torsional mode propagates unbiased and the measured speed agrees within two standard deviations with the reference speed. The analytic solution^34^ taking the geometry of the cylinder in account predicts a measured flexural wave speed of 2 m/s, which is in accordance with the experimental finding. This demonstrates the validity of our transient approach to quantify shear wave speed.

### In vivo cardiac experiments

**Figure 7:**
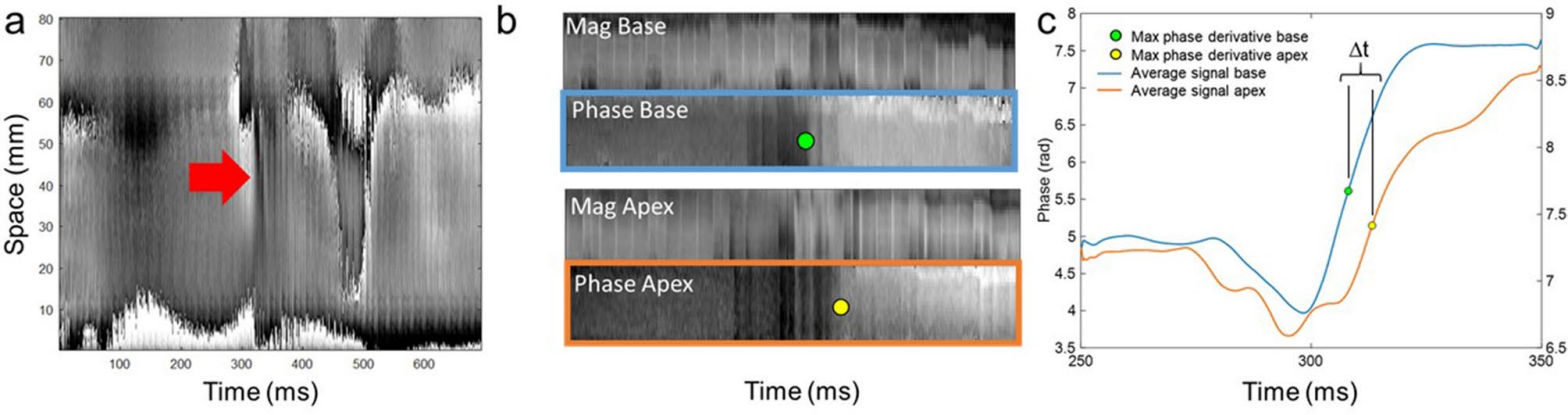
cNAVs positioned parallel versus perpendicular to the septum. (**a)** A single pencil beam positioned parallel to the septum generates a space–time waterfall plot, where space covers almost the entire length of the septum (80 mm). The plot clearly shows a perturbation at the time of aortic valve closure (~ 330 ms) with an inclination that yields a shear wave speed of about 5 m/s. For this experiment, the motion encoding was such that the torsional wave got selected. (**b)** The time-of-flight method uses two pencil beam volumes orientated perpendicular to the interventricular septum. Magnitude and phase images of the septum at the time of aortic valve closure. Space–time waterfall plots for the basal phase (Phase Base) and apical phase (Phase Apex) image in the septum show phase perturbations separated in time (green and yellow dot) indicative of the time-lapse needed for the shear wave to traverse the distance between both volumes. Note that space extends here only over the intersection between pencil beam volume and myocardium, i.e., approximately 10–20 mm. To enhance signal-to-noise ratio for subsequent processing steps, phase information is typically averaged over the myocardial wall direction. (**c)** Space averaged phase profiles for the two pencil beam volumes around aortic valve closure: blue = basal and orange = apical volume. The maximum phase derivative method yields two distinct time points (green dot and yellow dot) whose separation in time yields the time required for the wave to travel from one of the pencil beam volumes to the other. Since the spatial separation of the two volumes is known, the corresponding speed can be calculated directly.

Figure 7a shows the waterfall diagram for a healthy volunteer where the pencil beam was positioned parallel to the septum. A clear phase perturbation is visible around aortic valve closure approximately 320 ms after the beginning of the R-R cycle (red arrow). Here, the torsional wave mode was selected and a speed of about 4 m/s was measured. Figure 7b shows in vivo results for the time-of-flight approach using two navigators (denoted as Base and Apex), for both the magnitude and phase images. The vertical axes of the waterfall diagrams show the intersections between the navigator volumes with the septum; hence they represent space. The horizontal axes represent time. When considering the trailing edge of phase perturbations, the wave clearly first intersects with the ‘Base’ navigator volume, and then with the ‘Apex’ navigator volume. Phase-time profiles averaged over the thickness of the septum are shown in Fig. 7c. The maximum phase derivative method yields two distinct time-points (green and yellow dots) whose separation in time (Δt) provides the time required for the wave to travel from the 1st to the 2nd navigator. Note that the phase perturbations in Fig. 7b are quasi-vertical as the covered distance in space is very small. This is different to Fig. 7a which covers approximately 80 mm.

**Figure 8:**
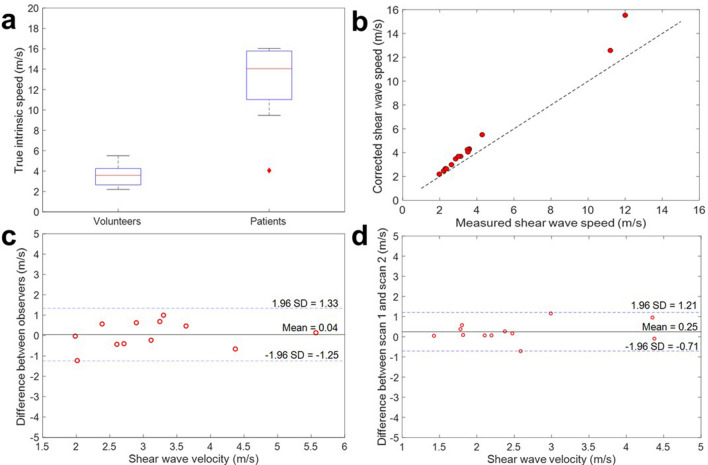
Application to healthy volunteers and cardiac patients. **(a)** Boxplot of true intrinsic wave speed obtained using the time-of-flight method in volunteers (left) and patients (right), indicating significant differences. The patient screened for but ultimately without cardiovascular pathology (red diamond) shows a speed in the range of the healthy volunteers. **(b)** Plot showing measured compared to shear wave velocity after correcting for bias caused by wall thickness for each participant. (**c)** Bland Altman plot for inter-observer variability between two operators. The mean difference between observer A and observer B was 0.04 m/s and 95% limits of agreement were − 1.25 and 1.33 m/s. Note that even with this variability, the difference in shear wave speed between volunteers and patients remains statistically significant. (**c)** Bland–Altman plot for interstudy variability between two scans. The mean difference between scan 1 and scan 2 for each individual was 0.25 m/s and 95% limits of agreement were – 0.71 and 1.21 m/s.

The boxplots of corrected wave speeds for healthy volunteers and patients are presented in Fig. 8a. Healthy volunteers showed a median wave speed of 3.6 (2.7–4.3)m/s. Patient #1 presented a shear wave speed of 4.1 m/s (red diamond), supporting the absence of any cardiovascular pathology. Consequently, this patient was excluded from overall statistical averages of the patient group. The remaining patients (n = 4) with confirmed cardiovascular pathology presented a median flexural wave velocity of 14.1 (11.0–15.8)m/s after correction, significantly higher (p = 0.0011) when compared to healthy volunteers. Figure 8b shows the measured shear wave velocity compared to the shear wave velocity after correction of the bias due to wall thickness for each participant. The mean wall thickness of the healthy volunteers was 11.5 mm (± 1.7 mm) and 16.0 mm (± 4.9 mm) for patients.

As seen in Fig. 8c, the inter-observer analysis using the Bland–Altman method resulted in a mean difference of 0.04 m/s between observer A and observer B, with 95% limits of agreement between -1.25 and 1.33 m/s.

The interstudy variability is shown in Fig. 8d. Here the mean difference in shear wave velocity between scans is plotted after performing the same scan twice in the same individual. Results show a mean interstudy difference of 0.25 m/s, with 95% limits of agreement between − 0.71 and 1.21 m/s. Both the inter-observer and the interstudy variation fall far below the observed differences between healthy volunteers and patients, with an average difference between the patient and control groups of over 10 m/s, confirming that this method has the potential to distinguish healthy volunteers from patients.

## Discussion

In this translational proof-of-concept study, we have successfully designed, developed, validated and applied a transducer-free method to measure LV septal myocardial shear wave velocity in healthy volunteers and patients using transient magnetic resonance elastography (cNAV-tMRE). The new sequence provides a tool to estimate in vivo myocardial tissue shear wave velocity non-invasively by utilizing naturally generated shear waves caused by aortic valve closure.

In initial experiments, we used a single pencil beam positioned parallel to the direction of shear wave propagation (i.e., along the length of the LV septum). Numerical simulations using a realistic heart model^46^ indicate that waves do propagate preferentially from base to apex. However, for in vivo applications, this approach was not robust enough due to partial volume effects. Therefore, we developed a new positioning strategy that minimizes such effects by placing two pencil beams lying perpendicular to the LV septum and measuring the time-of-flight between them. This strategy improved the reproducibility and the overall variability of left ventricular analysis, demonstrating minimal inter-observer variability in shear wave speed analysis and an overall interstudy variability that fell far below the variation in values between the healthy volunteer and patient cohorts.

Magnetic resonance and ultrasound elastography are modalities typically used to image the propagation of shear waves in biological tissues^47^. Recent studies have reported myocardial stiffness expressed in kilopascal (kPa) instead of shear wave velocity^11,30,48^. Fundamentally, in the absence of any attenuation effects, the stiffness is obtained from the square of the shear wave speed. As shown by both simulation and phantom data, the shear wave speed can be strongly biased by geometrical constraints, *i.e.*, wave guidance effects^21^, and consequently by the frequency at which the wave is generated since the modes become geometrically dispersive. Hence, these parameters need to be strictly controlled to allow for correction of potential biases. Assuming negligible attenuation, our healthy volunteers would have an approximate myocardial stiffness of about 13 kPa and the patients in the range of about 200 kPa. In the case of elevated viscosity, the simple conversion formula does not hold anymore. As viscosity is not measured, for this study we have therefore exclusively reported shear wave speed.

The phantom study indicated the ability to utilize the torsional wave to obtain unbiased shear wave speeds. Unfortunately, in vivo data show that the torsional mode often yields too low a signal to be exploitable for reliable wave speed analysis. This is most likely due to the predominantly longitudinal direction of mechanical excitation originating from the valve closure. We therefore based our in vivo analysis on the flexural mode originating from the longitudinal excitation. Aortic valve closure occurs over a time period of approximately 5 ms or less^35^, thus for this study, the frequency range of the generated shear wave was assumed to be between 200 and 300 Hz. Studies providing the frequency spectra of acoustic measurements of aortic valve closure in vivo show a peak frequency between 200 and 300Hz^36–38^, supporting this assumption. Using this, we determined an empirical correction factor for calculating unbiased shear wave speed based on known wall thickness and measured apparent shear speed. This is feasible since the pulse sequence enables selection of individual propagation modes and for this study, the flexural mode was chosen. Other available approaches often do not select a single wave mode and therefore cannot easily correct for the complex superposition of the individual biases. This issue could explain the differences between our values and those found in the literature, which are typically lower and around 1–2 m/s^11,19^.

Another potential reason for the higher wave speeds found in this analysis could be due to the specific timing of our measurement within the cardiac cycle. Myocardial stiffness changes during the cardiac cycle due to levels of filling and contraction of the ventricle. Aortic valve closure takes place at the end of systole/beginning of diastole, meaning the ventricle is still (partially) contracted and will provide a higher wave speed than studies performed in diastole. A numerical estimation of expected stiffness can be calculated based on the Laplace Law^49^. It assumes that myocardial stiffness = intraventricular pressure  ×  radius/(2 × wall thickness  ×  strain). In healthy subjects, this would lead to an end-systolic stiffness of ~ 30 to 40 kPa assuming an intravascular pressure of 13.3kPa^50^, a radius of 1.5cm^51^, a systolic wall thickness of 1.2cm^52^, and a strain 0.2–0.3^51^. This is in the order of magnitude of the stiffness values we reported in healthy volunteers and about one order of magnitude higher than values reported in other studies^11,19^.

Clear advantages of MR over ultrasound-based elastography methods are the ability to quantify shear wave propagation at any depth within the body and apply motion encoding in any arbitrary direction. Classical MRE using an external mechanical transducer has previously been described as a tool to non-invasively assess the viscoelastic properties of soft tissue by imaging the propagation of shear waves^53,54^. This technique has been extensively used for clinical evaluation of breast and liver lesions amongst other tissues, but its use for assessing cardiovascular disease and local stiffness of the left ventricle is currently not a routinely used diagnostic method in clinical settings. CMR is an ever increasingly used utility in the diagnostic workup of cardiac patients. However, as scan time is considerably more expensive than other imaging modalities, any proposed additional sequence must be fast and easy to incorporate. Currently available MRE techniques require an external transducer to generate shear waves in the desired tissue, impeding clinical implementation due to a number of limitations. These include the need for additional equipment within the MRI facility, additional time required for patient preparation with correct positioning of the transducer, altered ECG-lead placement to ensure appropriate triggering during the (clinical) scan and finally, the potential discomfort for patients relating to the use of the transducer. These limitations are overcome by the proposed method which should facilitate translation into clinical scanning protocols if future studies show the method to have added clinical value.

The difference in shear wave speed of about 10 m/s found in our analysis between healthy volunteers and cardiac patients is larger than initially anticipated. While shear wave velocity is expected to be larger in patients due to the underlying cardiac pathology, it is also possible that the myocardium takes longer to fully relax in patients, meaning that part of the observed elevated shear wave velocity in our experiments could originate from delayed relaxation effects. While this certainly represents a confounding aspect, it also offers the opportunity to identify subjects with a delayed cardiac relaxation. Furthermore, while Villemain et al*.*^11^ determined that age was the only physiological parameter associated with myocardial stiffness in a multivariable regression model using ultrasound elastography, associations between shear wave velocity and physiological parameters were not investigated in the current study. Parameters such as blood pressure could be a confounding aspect modifying myocardial stiffness. At present, we cannot distinguish if the increase in shear wave velocity is due to delayed relaxation, hypertension, or other diseases, but it is always related to cardiac disease. In further research, it could be beneficial to also assess whether mitral valve closure can be used for determining shear wave velocity as well as the aortic valve closure, as this would provide measurements for two separate cardiac phases.

It is worthwhile mentioning that the wave dispersion relationships we are using are derived for a hollow cylinder with stress free boundary conditions^34^. As the septal wall is buffered by blood on both sides, one may expect viscoelastic effects that could modify wave speed and amplitude in the left ventricle. Certainly, the amplitude of the radial displacement is impacted^55^ in our conditions^56^, but the wave speed remains basically unaffected for our geometry^57^. We therefore assume that septal wall buffering plays a minor role given our current level of precision.

For comparison of the cNAV-tMRE method to steady state MRE, we performed phantom experiments at 140 Hz. Measurements at higher frequencies were not feasible due to duty cycle limitations. While this could be seen as a limitation of this study, literature shows that variation and bias in shear wave velocity measured actually decreases as the frequency increases^58^. This is due to a decrease in wavelength, causing the wave to be less effected by spatial constraints, which in turn leads to less geometrically induced dispersion of the wave. While excitation frequency is known to influence velocity, the ability of this method to measure the velocity is not influenced by the excitation frequency.

A general limitation of our method is that it only functions for subjects in sinus rhythm, as data from multiple cardiac cycles are combined to provide sufficient temporal resolution to resolve the wave propagation. Also, as in many cardiovascular MRI sequences, combining data from multiple breath-holds and cardiac cycles could lead to the introduction of motion artefacts. In this study this effect is minimized by using a short NAV acquisition shot and short readout. Analysis of the hitherto acquired data has not been hindered by motion artefacts, however further studies will be needed to confirm this is not an issue in larger patient cohorts. It is yet to be determined if the presence of aortic valve disease, chiefly significant aortic stenosis and/or left ventricular outflow tract obstruction will have an impact upon the generation, propagation, and amplitude of shear waves within the LV septum and therefore preclude subsequent analysis. Another consideration requiring additional thought is that by acquiring transient motion from within only the LV septal wall, our sequence will have limited potential in the assessment of pathology that tends to favor the inferior and/or lateral walls of the left ventricle such as myocarditis or right coronary and circumflex territory infarcts. However, robust and well-established clinical validation of late gadolinium enhancement and T2-weighted imaging sequences in these types of pathology within the literature, would suggest this is not likely to present significant or clinically relevant drawbacks. Furthermore, one disadvantage of our method over previous cardiovascular MRE methods is that the cNAV-tMRE sequence does not produce an anatomical image and only provides a single outcome, namely shear wave velocity.

## Conclusion

We have demonstrated that it is feasible to measure myocardial shear wave velocity in vivo by measuring the speed of shear waves generated by aortic valve closure and without recourse to an external transducer. We have observed consistent values for healthy volunteers that differ significantly from those of our small cohort of patients. Results show a significantly higher myocardial shear wave speed in patients with cardiovascular pathology when compared to healthy volunteers. The large difference is thought to be due to both pathophysiological processes, as well as delayed relaxation effects. cNAV-tMRE may therefore be of diagnostic value and may provide in vivo insights into biomechanical changes which give rise to heart failure. Further work is required to establish its utility in clinical practice.

## Supplementary Information

Supplementary Figure S1.

## Data Availability

The datasets used and/or analyzed during the current study are available from the corresponding author on reasonable request.
