# Supplementary material for: Shear wave cardiovascular MR elastography using intrinsic cardiac motion for transducer-free non-invasive evaluation of myocardial shear wave velocity
Source: Sci Rep. 2021 Jan 14;11:1403. doi: 10.1038/s41598-020-79231-z (PMC7809276; doi:10.1038/s41598-020-79231-z)
Supplement: Supplementary file 1 — Supplementary Figure S1. [file 41598_2020_79231_MOESM1_ESM.docx]

# Supplementary material for “Shear wave cardiovascular MR elastography using intrinsic cardiac motion for transducer-free non-invasive evaluation of myocardial shear wave velocity.”

Marian Amber Troelstra, Jurgen Henk Runge, Emma Burnhope, Alessandro Polcaro, Christian Guenthner, Torben Schneider, Reza Razavi, Tevfik F Ismail, Jordi Martorell, Ralph Sinkus

**Supplementary Figure 1. Shear wave velocity measured via 3D MRE versus cNAV-tMRE with varying excitation frequencies.**

Using a gel exhibiting a shear wave velocity of approximately 1 m/s, propagation speed was measured using both a 3D MRE sequence and the cNAV-tMRE sequence, while varying the excitation frequency between 100 and 400 Hz. Shear wave speed for 3D MRE was 0.85, 1.01, 1.05 and 1.08 m/s at 100, 200, 300 and 400Hz respectively. cNAV-tMRE provided a shear wave speed of 0.88, 0.94, 1.00 and 1.00 m/s at 100, 200, 300 and 400Hz respectively. Mild dispersion was observed at lower frequencies, however, shear wave velocities between the methods were effectively identical with any small differences all being within the realms of measurement error.
